# Regulation and function of elF2B in neurological and metabolic disorders

**DOI:** 10.1042/BSR20211699

**Published:** 2022-06-20

**Authors:** Filipe M. Hanson, Rachel E. Hodgson, Madalena I. Ribeiro de Oliveira, K. Elizabeth Allen, Susan Gerarda Campbell

**Affiliations:** Biomolecular Sciences Research Centre, Industry and Innovation Research Institute, Sheffield Hallam University, Sheffield, S1 1WB, U.K.

**Keywords:** cellular targeting, eukaryotic gene expression, Protein synthesis, stress response, Translational Control

## Abstract

Eukaryotic initiation factor 2B, eIF2B is a guanine nucleotide exchange, factor with a central role in coordinating the initiation of translation. During stress and disease, the activity of eIF2B is inhibited via the phosphorylation of its substrate eIF2 (p-eIF2α). A number of different kinases respond to various stresses leading to the phosphorylation of the alpha subunit of eIF2, and collectively this regulation is known as the integrated stress response, ISR. This targeting of eIF2B allows the cell to regulate protein synthesis and reprogramme gene expression to restore homeostasis. Advances within structural biology have furthered our understanding of how eIF2B interacts with eIF2 in both the productive GEF active form and the non-productive eIF2α phosphorylated form. Here, current knowledge of the role of eIF2B in the ISR is discussed within the context of normal and disease states focusing particularly on diseases such as vanishing white matter disease (VWMD) and permanent neonatal diabetes mellitus (PNDM), which are directly linked to mutations in eIF2B. The role of eIF2B in synaptic plasticity and memory formation is also discussed. In addition, the cellular localisation of eIF2B is reviewed and considered along with the role of additional *in vivo* eIF2B binding factors and protein modifications that may play a role in modulating eIF2B activity during health and disease.

## Introduction

Eukaryotic cells are constantly exposed to a multitude of stress conditions, and the capacity to respond and adapt to this ever-changing environment is a driving factor behind our evolutionary success. The fundamental response to stress requires a rapid alteration of gene expression, to promote cell survival. However, if the stress is sustained or the stress cannot be overcome, this alteration of gene expression can induce cell death. Although transcriptional responses are crucial for controlling changes in gene expression, regulation at the translational level often allows for a faster response which permits immediate adaptation.

Translation can be divided into three main stages: initiation, elongation and termination. While regulation occurs at all stages of translation, the initiation stage is generally regarded as the rate limiting step and thus critical for ensuring efficient protein synthesis. This regulation is tightly controlled via the activity of the eukaryotic initiation factor 2B (eIF2B), which coordinates the ability of cells to maintain cellular proteostasis during both health and disease. This targeting of eIF2B is controlled via the coordinated activation of a series of signalling cascades collectively known as the integrated stress response, ISR. Dysregulation of the ISR pathway has been implicated in the pathological mechanisms of a broad spectrum of clinical conditions. This article focuses on the role of eIF2B in a number of these conditions, for a wider review the authors direct the readers to the excellent review by Costa-Mattioli & Walter [1]. The central role of eIF2B in synaptic plasticity and cognitive function has been elucidated by many research groups [2–4]. The development of the eIF2B GEF activity enhancers ISRIB and 2BAct [5,6] has shown the therapeutic benefit of the modulation of eIF2B activity in a range of cognitive disorders, as discussed below. Loss-of-function mutations in the EIF2B1-5 genes are associated with the rare, frequently fatal neurological disorder, leukoencephalopathy with vanishing white matter (VWMD) [7]. In this review, we discuss how VWMD mutations within eIF2B lead to phenotypic changes in specific cell types. We also review studies of cellular localisation of eIF2B subunits, an area which may further functional characterisation of pathogenic VWMD missense mutations. Finally, we discuss the role of eIF2B in the pathogenic mechanism of permanent neonatal diabetes mellitus (PNDM), a heterogeneous group of conditions diagnosed before the age of 6 months.

## eIF2 and eIF2B in translation

**Figure 1:**
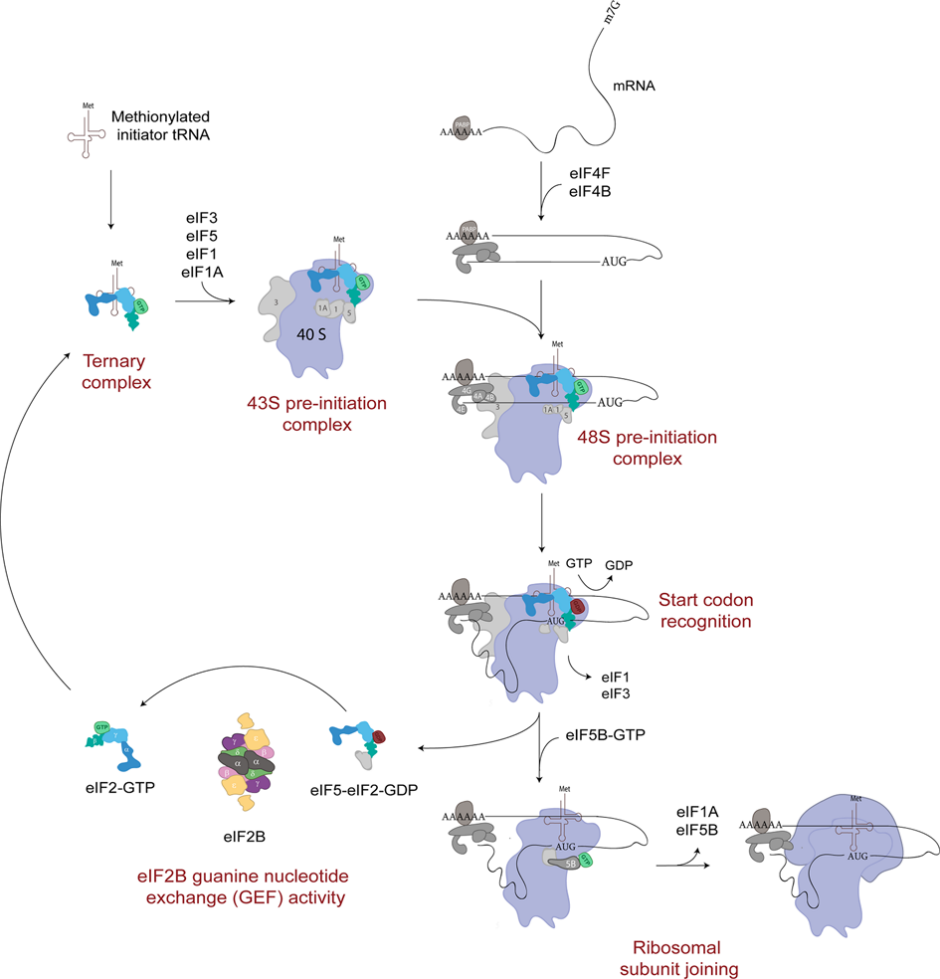
Translation initiation pathway A ternary complex formed of eIF2-GTP and a methionylated initiator tRNA is recruited to the 40S ribosomal subunit by various eIFs to form a 43 S PIC. Facilitated by other eIFs, the 43 S PIC is loaded onto a target mRNA molecule and scans the mRNA sequence for a start codon. Upon recognition of a start codon, eIF2-GTP is hydrolysed and released in complex with eIF5. eIF5B accommodates the binding of the 60S ribosomal subunit to the 40S subunit forming the elongation ready 80S ribosome. eIF2- GDP-eIF5 is recycled to eIF2-GTP by eIF2B.

Central to the regulation of translation initiation is the availability of sufficient levels of Met-tRNA_i_, which is delivered to the 43S preinitiation complex (PIC) through interaction with the GTP-binding protein eukaryotic initiation factor, eIF2 to form the eIF2-GTP.Met-tRNA_i_ ternary complex, (TC) (Figure 1). A detailed review of translation initiation is out of the scope of this review and the authors direct the readers to the following review articles [8,9]. In addition to the TC, the 43S PIC contains the small ribosomal subunit (40S) bound by the initiation factors eIF1, eIF1A and eIF3 (Figure 1) [9]. The 43S PIC scans the mRNA until a start codon is detected forming the 48S PIC. At this timeeIF2-GTP is hydrolysed via the activity of the GTP-activating protein, eIF5 and the inactive eIF2-GDP, which has a much lower affinity for Met-tRNA_i_ is released still associated with eIF5 (Figure 1) [9,10]. Key to the continued success of translation initiation is the recycling of the released eIF2-GDP to active eIF2-GTP and the recruitment of Met-tRNAi to form new TC. This recycling role is carried out by the guanine nucleotide exchange factor, eIF2B (Figure 1). As eIF2-GDP is released from the 48S PIC as an eIF2-GDP/eIF5 complex this enables eIF5 to function as a GDP dissociation inhibitor (GDI) inhibiting any spontaneous exchange activity [11]. In yeast, eIF2B has also been shown to act as a GDI displacement factor (GDF) to release eIF2-GDP from eIF5 [12,13]. The exact mechanism for how new active TC is formed is still unclear however recent evidence suggests that a complex interplay between eIF2B, eIF5 and Met-tRNAi can influence the stability of TC (Figure 1) [14].

While the eIF2-GTP is critical for recruiting Met-tRNAi to the start codon, alternative factors, such as eIF2A have been identified which can also promote this recruitment albeit when eIF2 levels are low and can result in targeted translation of specific mRNAs. A detailed analysis of the role of eIF2A and other alternative factors is outside the scope of this review, but the authors direct the readers to an detailed review on the topic [15].

**Figure 2:**
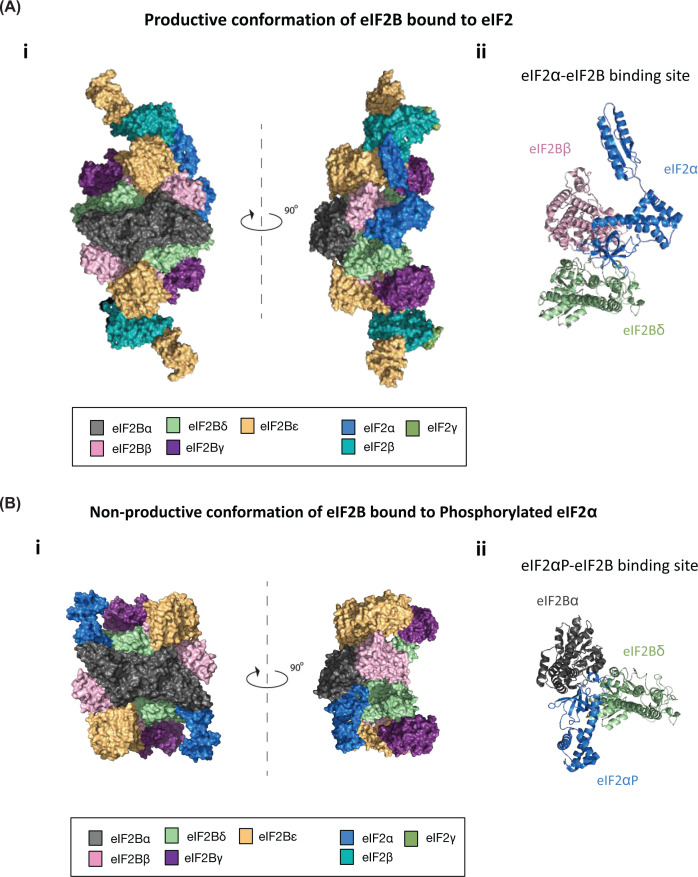
CryoEM structures resolved for eIF2B bound to non-phosphorylated or phosphorylated eIF2 (**A**) Orthogonal views of (i) decameric eIF2B in its productive conformation bound to a non-phosphorylated eIF2 heterotrimer shown in surface representation, (ii) with a cartoon representation of the eIF2α-eIF2B binding site (PDB 6O81 [27]). (**B**) Orthogonal views of (i) decameric eIF2B in its non-productive conformation bound to two phosphorylated eIF2α subunits shown in surface representation, (ii) with a cartoon representation of the phosphorylated eIF2α-eIF2B binding site (PDB 6O9Z [27]).

eIF2B exhibits a greater level of complexity within its quaternary structure. It is composed of five nonidentical subunits, encoded by the genes EIF2B1–5 and termed eIF2Bα through to ε, respectively (Figure 2Ai). The γ and ε subunits catalyse the guanine nucleotide exchange activity of eIF2B, whereas the α, β and δ subunits are required to regulate this activity in response to various cellular signals [16–19]. While eIF2Bɛ on its own can carry out exchange activity (the C-terminal domain of eIF2Bɛ facilitates binding of eIF2 while the HEAT domain can catalyse eIF2 nucleotide exchange), the rate of this exchange is greatly enhanced through joining of the other eIF2B subunits [20].

The eIF2B regulatory subunits are responsible for modulating levels of eIF2B activity, dependent on the cellular environment. In response to conditions of cellular stress, specific eIF2 kinases (discussed in more detail later) phosphorylate the α subunit of eIF2 (p-eIF2α), converting eIF2 from a substrate to a competitive inhibitor of eIF2B GEF activity [21,22]. eIF2Bα in particular is required to facilitate this inhibition however detailed mutational analysis of eIF2Bβ and eIF2Bδ highlights that these subunits also contribute [17,23–29]. Precise expression levels of the eIF2B subunits within the cell is crucial for tight control of eIF2B activity [30]. Wortham et al*.* demonstrated that stable expression of eIF2Bε requires stoichiometric expression of eIF2Bγ and that stable expression of eIF2Bδ requires stoichiometric expression of eIF2Bβ. In the absence of co-stoichiometric expression eIF2Bε and δ subunits are ubiquitinated, targeting them for ubiquitin-proteasome degradation.

Prior to 2014, eIF2B was believed to be a pentameric complex comprises one copy of each of its subunits; however, mass spectrometry analysis has revealed that eIF2B exists as a decameric complex, comprises two copies of each of its subunits [31,32]. The first crystal structure of eIF2B was solved from *Schizosaccharomyces pombe* and revealed a central core composed of two copies of each of the regulatory subunits (α, β and δ), flanked at opposite sides by a heterodimer of the catalytic subunits (γ and ε) (Figure 2Ai) [33]. It is believed that these decameric eIF2B complexes form through the stabilisation of two eIF2B(βδγε) tetramers with one eIF2Bα homodimer [32–34].

A number of cryo EM structures for mammalian and yeast eIF2B have now been solved together with the substrate eIF2 and the phosphorylated form of eIF2α [35–41]. These structures have provided detailed observations of structural rearrangements that take place through the binding of p-eIF2α to eIF2B (Figure 2A,B). In its productive or apostate conformation, eIF2γ (which contains the GTP binding site) binds primarily to eIF2Bε, and the α subunit of eIF2 makes contact with the regulatory subunits eIF2Bβ and δ (Figure 2Aii) [35–40]. However, when eIF2α is phosphorylated, structural changes are observed which are critical for the inhibition of eIF2B activity. The N-terminal domain of p-eIF2α now makes contact between the eIF2Bα and eIF2Bδ subunits which induces a conformational change and inhibits the interaction of eIF2γ with the eIF2Bε subunit (Figure 2Bii). This inhibitory structural rearrangement has been termed the non-productive conformation (Figure 2B).

Detailed reviews of these structures have been previously published and the authors direct the readers to these [42,43].

## eIF2B and the integrated stress response

**Figure 3:**
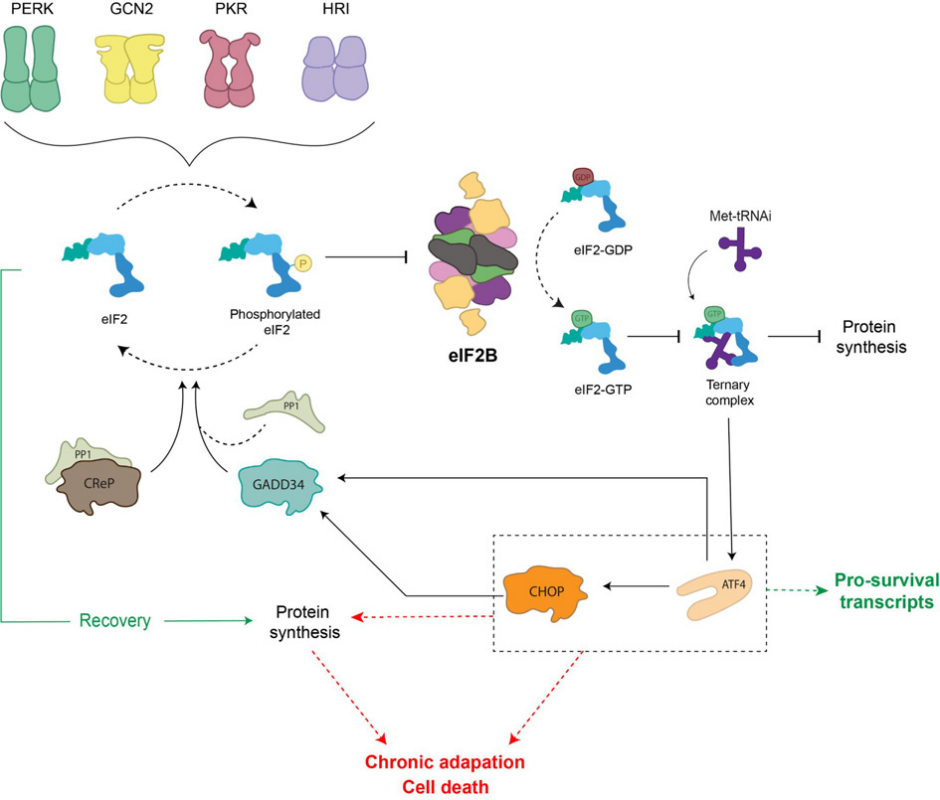
Activation of the ISR pathway In response to various cellular stress stimuli eIF2α kinase molecules are activated through dimerisation. eIF2α kinases phosphorylate the α subunit of eIF2. In its phosphorylated form, eIF2 is a competitive inhibitor of eIF2B activity preventing replenishment of eIF2-GTP within the cell. This leads to inhibition of global protein synthesis while the translation of specific stress response mRNAs, including ATF4, is up-regulated. During episodes of acute ISR, ISR effectors are able to restore homeostasis and ATF4-mediated activation of CHOP induces the transcription of GADD34 to promote dephosphorylation of eIF2α. In cases where ISR effectors are unable to restore homeostasis, the cell transitions into a chronically activated ISR. Protein synthesis is restored via an eIF2B independent mechanism and ATF4-mediated activation of CHOP promotes proapoptotic gene expression.

As mentioned above, the eIF2Bα, β and δ subunits are required to regulate eIF2B activity in response to various cellular signals via recognising and interacting with p-eIF2α, thereby causing structural rearrangements of eIF2B which inhibit its GEF activity. This phosphorylation event is carried out by four eIF2α kinases which when activated via specific stresses, phosphorylate the same single serine residue (S51) of eIF2α (Figure 3) [44]. Collectively, the function of these stress-sensing kinases and the subsequent effect on eIF2 and eIF2B activity is known as the integrated stress response, ISR [45,46]. These kinases are termed: GCN2 (general amino acid control nonderepressible 2), PKR (protein kinase RNA-like), PERK (protein kinase RNA-like endoplasmic reticulum kinase) and HRI (heme-regulated inhibitor) (Figure 3). Recently, it has been suggested that there may be a fifth kinase (MARK2) which can respond to proteotoxic stress and phosphorylate eIF2α [47]. While the eIF2α kinases share extensive homology in their catalytic domains (a dimerisation interface, crucial for kinase activation and catalytic function), each kinase has an unique regulatory domain which allows for activation of the ISR by a range of cellular stresses [48–52]. GCN2 is activated in response to amino acid deprivation in order to reduce the cellular demand for amino acids and is the only kinase conserved from yeast to mammalian cells [53]. PKR was thought to be activated primarily by double-stranded RNA (dsRNA) during viral infection; however, it can also be activated in response to oxidative stress, serum deprivation and more recently has been associated with the activation of proinflammatory signalling in response to pathogens [54–55]. Interestingly, while dsRNA can activate PKR directly, the activation of PKR by other stresses relies on its interaction with the protein activator of PKR (PACT) 1 [56]. PERK is principally activated in response to endoplasmic reticulum (ER) stress, commonly caused by the accumulation of unfolded proteins in the ER. PERK activation alleviates this stress by decreasing the level of proteins localised to the ER [57,58]. HRI while originally thought to protect erythroid cell against toxic globin aggregates, it can also be activated in non-erythroid cells in response to arsenite induced oxidative stress and more recently has been shown to play an important role in protecting cells from proteotoxicity [59–61].

The phosphorylation of eIF2α caused by these kinases converts eIF2 into a competitive inhibitor of eIF2B activity [62,63]. This results in a depleted level of eIF2-GTP within the cell, thereby decreasing levels of ternary complex which leads to a global translational attenuation of protein synthesis (Figure 3). In a recent review, the molar concentrations of a number of translation initiation factors from both yeast and HeLa cells were collated [9]. Here, it was shown that cells have approximately 3- to 5-fold less eIF2B complexes than eIF2; this highlights the significance of regulating eIF2B activity as even a minimal level of p-eIF2α can decrease eIF2B activity.

Paradoxically, during this time the translation of a number of stress responsive proteins is upregulated. The translation of these mRNAs is most commonly regulated by the presence of upstream open reading frames (uORFs) in their 5' UTR [64]. uORFs are generally inhibitory for the translation of a mRNA transcript under normal cellular conditions; however, during episodes of cellular stress, they can promote the translation. Key to the ISR regulation is the mRNA for the transcription factor, Activating Transcription Factor 4 (ATF4). ATF4 mRNA is ubiquitously expressed; however, under normal cellular conditions protein levels are low. ATF4 mRNA contains two uORFs [64]. Under normal cellular conditions, the first uORF which encodes a short polypeptide (three amino acids) is translated. This up-stream uORF acts as a positive element allowing ribosomes to scan and initiate translation at the next uORF. The second uORF sequence (59 amino acids) overlaps with the coding sequence of ATF4 in an out-of-frame manner, and, therefore, the translation of uORF2 inhibits the translation of ATF4 [64]. However during stress when eIF2B activity in inhibited and TC levels are low the rate of re-initiation at the second uORF decreases thereby increasing re-initiation of the ATF4 ORF. Increased ATF4 expression can activate prosurvival mechanisms within the cell through a number of different pathways. This mechanism is highly conserved and in yeast the transcription factor Gcn4p has 4 uORFs which regulate its expression upon ISR activation [65]. More detailed reviews of uORFs and their role in translational control are available [66].

If ISR signalling leads to the restoration of cellular homeostasis, ATF4-mediated activation of the transcription factor C/EBP homologous protein (CHOP) can contribute to the restoration of global translation. CHOP induces the transcription of growth arrest and DNA damage inducible protein (GADD34), an eIF2α phosphatase regulatory subunit which binds to PP1c to allow the regulated dephosphorylation of eIF2α (Figure 3) [67]. In cases of severe cellular stress where the pro-survival mechanisms induced by the ISR are unable to restore homeostasis, the ISR promotes programmed cell death signalling (Figure 3). As the ISR provides a central network for maintaining cellular homeostasis, dysregulation of ISR signalling has numerous pathological consequences and has been linked to conditions such as: cancer, diabetes, cardiovascular disease and neurodegeneration [1]. Thus, pharmacological modulation of the ISR has become a key area for therapeutic research. A detailed appreciation of these targets and therapeutics has been reviewed elsewhere [46,68], so only a few will be highlighted and discussed in this review.

Of particular interest is the small molecule known as Integrated Stress Response InhiBitor (ISRIB) [69]. ISRIB is a direct activator of eIF2B and was originally identified following a screen of compounds that could reduce the ISR [71]. Recent analysis of ISRIB binding to eIF2B and its competitive relationship with p-eIF2α has provided insight into the mode of action of ISRIB [37,38]. ISRIB binding has been shown to favour stabilisation of the productive form of eIF2B thereby making eIF2B relatively resistant to the inhibitory effects of p-eIF2α [37,38]. ISRIB does not affect the level of p-eIF2α but rather through interactions with eIF2Bβ and δ subunits; ISRIB promotes stabilisation of two tetramers to form an eIF2B(βδγɛ)_2_ octamer that favours eIF2Bα homodimer binding thus promoting decameric formation and enhancing eIF2B GEF activity [71,34–37]. Interestingly, ISRIB treatment has been shown to enhance cognition in aged mice while also reversing cognitive deterioration associated with neurodegeneration and traumatic brain injury [72–77]. A number of derivatives of ISRIB have been produced which show similar effects of cognitive enhancement and are currently in phase 1 clinical trials [77,79].

## eIF2B in acute and chronic activation of the integrated stress response

It has been commonly reported that an acute and transient activation of the ISR prompts a global reduction of translation and induction of genes involved in supporting cellular recovery to regain homeostasis. In contrast, transition to a chronically activated ISR is widely reported as adaptive to prolonged stress, ultimately pro-apoptotic when cells are unable to overcome sustained stress with pathological consequences [80–82]. One of the best studied mechanisms of ISR-induced cell death involves ATF4-mediated activation of and interaction with CHOP [83–86]. CHOP has been shown to induce apoptosis via a number of mechanisms including the repression of anti-apoptotic proteins [87] and up-regulation of death receptors [88]. Hence, ATF4 and CHOP have intricate roles in the ISR and function to produce tailored responses, both pro- and anti-survival dependent on the cellular stress stimuli (Figure 3).

The role of eIF2B during the acute phase of the ISR has been defined and reviewed elsewhere [46]. eIF2B activity is inhibited to prevent ternary complex formation and allow ATF4-induced reprogramming, followed by restoration of eIF2B activity and protein synthesis hence hallmarking the termination of the acute ISR. However, its specific role during chronic stress is still unclear. Interestingly, as a cell transitions into a chronically activated ISR, it has been suggested that the restoration of protein synthesis does not require the recovery of eIF2B activity (Figure 3) [89]. Guan et al*.* suggest that a sustained reduction in eIF2B activity during chronic ER stress results in transcriptional and translational remodelling which is dependent on the activation of PERK and mediated by eIF3 [89]. In addition, pharmacological activation of eIF2B in chronic ER-stressed human fibroblast cells synergistically reduced viability by triggering caspase-3/7-mediated apoptosis [70], suggesting that the cellular response to eIF2B activation is dependent on the level and duration of ER stress.

Cellular adaptation to stress may correlate with levels of p-eIF2α and cell type-specific demand of global protein synthesis. Secretory cells, such as pancreatic β-cells, require basal p-eIF2α to prevent oxidative damage and rapid unburden of ER load [90–92]. Increased levels of p-eIF2α, hence sustained eIF2B inhibition, sensitises β-cells to CHOP-mediated apoptosis [93]. In contrast, hypo-phosphorylation of eIF2α renders β-cells with uncontrolled translation and deregulated antioxidant levels, highlighting the narrow threshold of eIF2α modulation and eIF2B inhibition [94–96]. Therefore, the cellular control of eIF2B activity in the transition from acute to chronic stress is critical and allows them to adapt and survive the shift from a protective short-lived ISR to adaptation to chronic stress.

## eIF2B bodies and cellular localisation

**Figure 4:**
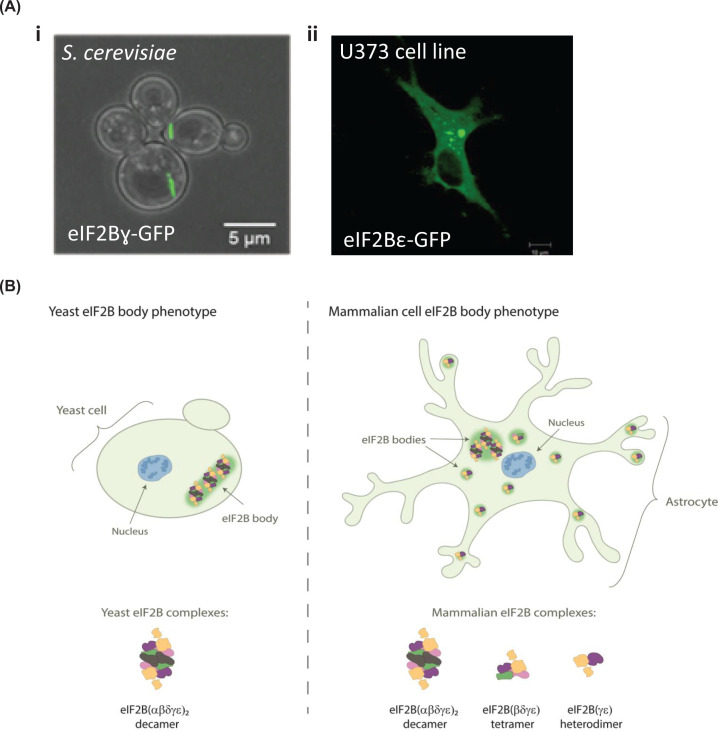
eIF2B bodies in yeast and mammalian cells (**A**) Confocal images of eIF2B bodies in (i) *S. cerevisiae* strain yMK880 (eIF2Bγ-GFP) (ii) a human U373 astrocytoma cell overexpressing eIF2Bε-GFP. (**B**) Schematic representation of decameric eIF2B(αβδγε)2 and eIF2B(βδγε) tetrameric and eIF2B(γε) heterodimeric subcomplexes that have been identified in yeast and mammalian cells localising to eIF2B bodies.

The cellular localisation of eIF2B has gained interest over the past two decades. In 2005, the Ashe group first identified cytoplasmic accumulations of eIF2B subunits in *Saccharomyces cerevisiae (S. cerevisiae)* and termed them eIF2B bodies [97]. Phenotypically each yeast was found to harbour one eIF2B body that formed a large filament shaped structure (Figure 4). These eIF2B bodies were distinct from accumulations of mRNA and other translation initiation factors, with the exception of the three subunits of eIF2. Given eIF2B’s role as a GEF for eIF2, eIF2B bodies were hypothesised to be sites of eIF2B GEF activity, required for translation initiation. Indeed, eIF2 was shown to dynamically localise to the eIF2B body, in a manner that correlated to eIF2B GEF activity within the cell [97]. A later study revealed that *S. cerevisiae* eIF2B bodies are motile within the cytosol and that inhibition of this motility is associated with the inhibition of translation [98]. More recently, deletion of eIF2Bα in yeast has been shown to disperse eIF2B bodies, suggesting that the decameric conformation of eIF2B is required for eIF2B body stability [99]. Interestingly, an eIF2Bα^V184D^ point mutation that does not destabilise the decameric conformation of eIF2B [100,33] and disrupts eIF2B body integrity in yeast [99]. In eIF2Bα^V184D^ mutant yeast, eIF2B localises to multiple small punctate foci, which have been termed microfoci. These microfoci were also observed in the presence of eIF2Bα gcd^−^ (gcn2 depressible) mutations, associating catalytic impairment and eIF2B body stability. Through combining SEM imaging and a subtomogram-averaging approach, Marini et al*.* have obtained a 3D model of eIF2B bodies in yeast upon energy depletion [101]. Comparing the volume of the eIF2B decamer, the authors hypothesise that eIF2B bodies are repeats of decameric units, stacked through contacts between eIF2Bε subunits of adjacent decamers. Further investigation into the stability and structural arrangement of eIF2B bodies may provide a greater insight into their function.

The physiological relevance of eIF2B body formation within yeast has been debated. A number of studies have demonstrated that eIF2B bodies form more readily under conditions of glucose starvation and cytoplasmic acidification [102–105]. These are conditions that promote translational inhibition and thus, as eIF2B bodies appear to be sites of GEF activity, an increased formation under these conditions appears counterintuitive. Norris et al*.* hypothesised that as different yeast strains respond differently to environmental stresses, eIF2B body formation may also vary between yeast strains [99]. In support of this it was found that, the percentage of cells with eIF2B bodies in two different strains of *S. cerevisiae* varied significantly under steady state growth. The percentage of cells with eIF2B bodies increased within both strains following amino acid starvation, but rather unexpectedly an increase was not observed during glucose starvation. Additionally, it may be interesting to speculate whether differences may also be explained by variations in molar concentrations of the eIF2 and eIF2B protein complexes across the different yeast strains which could influence the formation of eIF2B bodies [9,106]. Further investigation into strain specific stress responses and concentrations of eIF2 and eIF2B subunits may provide a greater insight into the driving force behind eIF2B body formation.

Although the mechanism that promotes eIF2B body formation in yeast remains unclear, in mammalian cells eIF2B bodies have been shown to form under normal culture conditions. Hodgson et al. showed endogenously expressed eIF2B and eIF2 subunits localise to discrete cytoplasmic foci [107]. Fluorescently tagged proteins were used to analyse the dynamics between eIF2B subunits and eIF2α within these foci. eIF2B was found to be a stable component, whereas eIF2 is mobile; shuttling through the body in a manner that correlates with cellular eIF2B GEF activity. Interestingly, the composition and function of these eIF2B bodies appears to be regulated by the ISR. Unlike in yeast where a single eIF2B body exists, mammalian cells contain a number of eIF2B bodies that vary in size and eIF2B subunit-makeup (Figure 4). A high degree of co-localisation for all five eIF2B subunits was observed in larger eIF2B bodies. As expected, upon acute ISR activation, the association of p-eIF2α with these bodies was increased and the movement of eIF2 decreased, suggesting impaired GEF activity. Regulatory eIF2B subunits were rarely observed to co-localise with smaller eIF2B bodies (made of predominantly γε subunits), and thus it was hypothesised that ISR induction would not impact on the GEF activity of these bodies (Figure 4). Intriguingly, the movement of eIF2 through these smaller bodies was found to increase upon ISR stimulation, suggesting that eIF2B GEF activity is increased. This was accompanied by an increased degree of co-localisation with eIF2Bδ, suggesting that novel eIF2B(δγε) subcomplexes potential form following ISR activation. These data suggest that pools of eIF2B subcomplexes exist in mammalian cells and may have a role in mediating cellular stress responses. Subcomplexes of eIF2B have previously been shown in mammalian cells through native mass spectrometry (MS) [32]. eIF2B(βδγε) tetramers, eIF2B(γε) heterodimers and eIF2B(α_2_) homodimers were identified but not eIF2B(δγε) subcomplexes. Previous studies have shown eIF2Bβ and δ subunits can heterodimerise through C-terminal domain interactions [108,109]. Furthermore, eIF2Bβ is required for stable expression of eIF2Bδ [30] and thus the observed co-localisation of eIF2Bδ, but not eIF2Bβ with small eIF2B bodies in stressed cells is rather intriguing. Although Wortham et al*.* did not identify eIF2B(δγε) subcomplexes by native MS [32], application of high collision energy to disrupt the eIF2B(βδγε) tetramer led to the dissociation of eIF2Bβ but not eIF2Bδ from the complex, suggesting that eIF2Bδ can interact with eIF2Bγε in the absence of eIF2Bβ. Native MS analysis of eIF2B complexes during ISR stimulation could provide further insight into the existence of an eIF2B(δγε) subcomplex.

## eIF2B post-translational modifications and interacting molecules

Post-translational modifications (PTMs) have been shown to influence eIF2B activity. Phosphorylation of eIF2Bε is crucial for eIF2B function [110]. Glycogen synthase kinase-3 (GSK3) catalyses phosphorylation of eIF2Bε at Ser535 and limits eIF2B activity [111]. Inhibition of GSK3 activity by insulin leads to Ser535 dephosphorylation and eIF2B activation. Two other phosphorylation sites at the C-terminus of eIF2Bε facilitate eIF2 binding [112]. Similarly, another study highlighted increased phosphorylation sites in yeast catalytic eIF2Bγ- and ε-, where the latter subunit showed phosphorylation site clustering located in the highly unstructured flexible loop connecting the ε/γ dimer [113]. eIF2 relies on PTM-mediated adaptable conformational changes according to its functional role [113]. Like eIF2, the subunits of eIF2B could use PTMs to switch between productive and non-productive conformations towards eIF2 binding, however the structural impact and the functional role of these PTMs in eIF2B activity remains largely unknown. Novel acetylation sites have also been identified in eIF2B. Acetylation has been broadly linked to the regulation of phosphorylation susceptibility of large protein complexes [114]. Interestingly, eIF2Bε remains largely absent of acetylation sites, whereas the eIF2B regulatory subunits reveal a remarkably conserved N-terminal acetylation site [113]. Such sites may have a role in stabilising complex formation as N-terminal acetylation prevents protein degradation [115]. On the contrary, N-terminally acetylated proteins can also be directed towards degradation [116]. Wortham et al*.* (2016) reported stoichiometric expression of eIF2B subunits is mediated by ubiquitin-mediated degradation [30]. A further understanding of the significance of these PTMs sites could uncover new regulation layers of eIF2B function.

In addition to PTMs, molecular interactions with eIF2B are critical for its function. eIF2α phosphorylation status defines the affinity fate of eIF2 to eIF2B [117] while eIF2-GDP is complexed with eIF5, to regulate GEF activity [11]. More recently, it has been shown that the binding of GTP to eIF2Bγ not only serves to provide a pool of available GTP but also enhances GEF activity [118]. Natural sugar metabolites bind to eIF2Bα_2_ dimers and promote eIF2B decameric formation [119]. In yeast, mutations in these sugar phosphate binding sites of eIF2Bα have been shown to disperse eIF2B bodies [100]. Viral proteins counteract translational shutdown by binding to host eIF2B and induce its productive state independently of p-eIF2α [120,121]. While some additional protein factors have been shown to bind to eIF2B [122,123], the contribution of other binding partners to eIF2B activity has not received much attention. Analysis of published data of protein–protein interactors of each eIF2B subunit curated by BioGRID (release 4.4.201) [124] is outlined in Table 1. The list of identified genes was annotated with respective slim GO terms through GOnet (update January 2020) [125]. This analysis has shown that there are many potential interactors of eIF2B, and these seem to share similar gene functions which go far beyond the scope of translation (Table 1). Future investigation of these interactors could provide new avenues of investigation for novel roles of eIF2B.

**Table 1:** Biological processes of eIF2B protein–protein interactors

Go term ID	GO term annotation	Number of genes involved per eIF2B subunit				
		eIF2Bα	eIF2Bβ	eIF2Bγ	eIF2Bδ	eIF2Bε
GO:0007165	Signal transduction	40	37	39	48	46
GO:0006810	Transport	34	38	31	30	43
GO:0048856	Anatomical structure development	40	42	37	43	42
GO:0002376	Immune system process	28	28	25	27	34
GO:0006950	Response to stress	35	26	36	28	33
GO:0030154	Cell differentiation	35	30	24	33	30
GO:0034641	Cellular nitrogen compound metabolic process	33	41	32	28	27
GO:0009058	Biosynthetic process	23	33	24	20	27
GO:0022607	Cellular component assembly	14	22	17	16	25
GO:0042592	Homeostatic process	16	20	17	18	24
GO:0006464	Cellular protein modification process	18	22	17	19	23
GO:0040011	Locomotion	12	14	10	14	21
GO:0015031	Protein transport	20	18	12	13	19
GO:0016192	Vesicle-mediated transport	13	16	11	11	19
GO:0065003	Protein-containing complex assembly	11	16	13	11	18
GO:0009056	Catabolic process	17	17	10	11	16
GO:0048870	Cell motility	9	10	8	11	16
GO:0007155	Cell adhesion	7	11	8	12	15
GO:0007267	Cell–cell signalling	11	11	11	12	15
GO:0008219	Cell death	9	9	9	9	15
GO:0044281	Small molecule metabolic process	6	12	12	8	14
GO:0055085	Transmembrane transport	8	11	9	9	13
GO:0044403	Symbiotic process	16	12	11	10	12
GO:0061024	Membrane organisation	11	13	11	9	12
GO:0006412	Translation	13	14	9	7	11
GO:0006629	Lipid metabolic process	6	8	6	5	11
GO:0000003	Reproduction	15	13	12	10	10
GO:0007049	Cell cycle	8	7	6	4	10
GO:0008283	Cell population proliferation	4	7	7	6	9
GO:0050877	Nervous system process	5	6	7	6	9
GO:0007010	Cytoskeleton organisation	2	2	4	4	9
GO:0051276	Chromosome organisation	6	11	10	9	8
GO:0051301	Cell division	4	2	3	2	8
GO:0000902	Cell morphogenesis	7	10	4	7	7
GO:0006259	DNA metabolic process	4	8	7	6	7
GO:0000278	Mitotic cell cycle	4	2	4	2	6
GO:0007568	Aging	4	4	3	4	5
GO:0006914	Autophagy	5	5	4	3	5
GO:0003013	Circulatory system process	6	4	5	5	4
GO:0034655	Nucleobase-containing compound catabolic process	5	7	2	5	4
GO:0022618	Ribonucleoprotein complex assembly	3	6	4	2	4
GO:0007034	Vacuolar transport	3	1	2	1	4
GO:0007005	Mitochondrion organisation	3	2	4	0	4
GO:0009790	Embryo development	5	7	7	8	3
GO:0006605	Protein targeting	7	7	3	4	3
GO:0006790	Sulfur compound metabolic process	3	3	1	2	3
GO:0021700	Developmental maturation	2	2	0	2	3
GO:0007059	Chromosome segregation	1	1	2	1	3
GO:0042254	Ribosome biogenesis	5	4	2	8	2
GO:0006397	mRNA processing	6	9	6	5	2
GO:0048646	Anatomical structure formation involved in morphogenesis	1	3	2	5	2
GO:0006457	Protein folding	1	2	1	2	2
GO:0034330	Cell junction organisation	1	2	2	2	2
GO:0040007	Growth	2	1	0	2	2
GO:0006091	Generation of precursor metabolites and energy	1	2	2	1	2
GO:0006913	Nucleocytoplasmic transport	2	2	2	1	2
GO:0030705	Cytoskeleton-dependent intracellular transport	1	3	2	1	2
GO:0140014	Mitotic nuclear division	0	0	1	0	2
GO:0051186	Cofactor metabolic process	2	2	2	2	1
GO:0005975	Carbohydrate metabolic process	0	0	0	1	1
GO:0007009	Plasma membrane organisation	1	2	0	1	1
GO:0006091	Extracellular matrix organisation	0	1	0	0	1
GO:0019748	Secondary metabolic process	1	0	0	0	1
GO:0043473	Pigmentation	0	1	0	0	1
GO:0051604	Protein maturation	1	0	0	1	0
GO:0006399	tRNA metabolic process	1	3	0	0	0
GO:0006520	Cellular amino acid metabolic process	4	2	2	0	0

Identification of genes involved in protein–protein interactions was performed and curated by BioGRID (release 4.4.201). Gene ontology classification was performed using GOnet (DICE database, build January 2020). The whole human repository annotation was used as reference set.

## eIF2B in synaptic plasticity and cognitive decline

**Figure 5:**
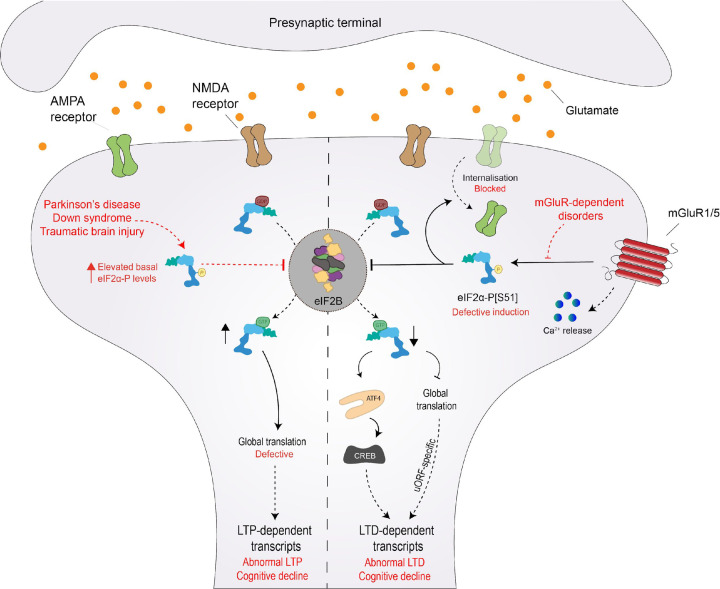
eIF2α phosphorylation and eIF2B modulation are key events in LTP- and LTD-dependent translation eIF2α phosphorylation status is a major switch of synaptic plasticity, dictating whether a given synapse undergoes long-term potentiation (LTP) or long-term depression (LTD). Suppression of eIF2α phosphorylation induces LTP by favouring new protein synthesis of ‘LTP proteins’. In contrast, increased levels of eIF2α phosphorylation drives the expression of LTD-specific proteins. Impairment of these synaptic changes triggers cognitive decline (highlighted in red), inherently associated with neurodegenerative disorders that bidirectionally affect levels of eIF2α phosphorylation.

eIF2B and the regulation of protein synthesis plays a key role in synaptic plasticity and cognitive function [2–4]. Synaptic plasticity can be defined by the activity of synaptic connections, which ultimately coordinates the basis of learning and memory storage. High activity strengthens synapses, prompting long-term potentiation (LTP), while low activity weakens it, resulting in long-term depression (LTD) [126]. Interestingly, the phosphorylation status of eIF2α and therefore eIF2B activity, can dictate the fate of a given synapse, either facilitating LTP or LTD (Figure 5). Synapses undergoing local reductions of p-eIF2α are predicted to be potentiated. Upon eIF2α phosphorylation, mRNA translation of ATF4 suppresses CREB, a major transcription factor of plasticity-relevant proteins (Figure 5) [127,128]. In support of this, mutant eIF2α heterozygous mice (eIF2α^+/S51A^) displayed improved LTP and long-term memory consolidation [2]. In contrast, LTD relies on increased levels of p-eIF2α. Prisco et al*.* (2014) elegantly reported that uORF-driven translation remodels expression of cell surface receptors at synapses required for mGluR-LTD (Figure 5) [129]. It is the current view that this modulation of the eIF2α phosphorylation status can be adjusted to support a given learning task. LTP-dependent paradigms, such as contextual fear conditioning, shifts synapses to repress p-eIF2α, while LTD learning programs, such as object-in-place learning, demands the regulated translation of transcripts containing uORFs [129,130].

As the hub of adaptability to learning and long-term memory storage, eIF2α phosphorylation, and thus modulation of eIF2B activity, has been studied in detail for cognition improvement. ISRIB, the eIF2B GEF activity enhancer, has been shown to attenuate p-eIF2α-dependent translational control without changes to eIF2α phosphorylation status *per se* or evidence of toxicity [74,131,132]. Indeed, eIF2B activation strengthens synaptic plasticity and memory consolidation in healthy rodents [70]. ISRIB also proved beneficial to counteract abnormally elevated levels of p-eIF2α and LTP-impairment in models of Parkinson’s [133], Down syndrome [74,134], traumatic brain injury [77], alcohol addiction [135] and drug abuse [136]. However, less is known of the impact of eIF2B activity modulation on LTD synapses which require p-eIF2α. Conflicting studies in Alzheimer’s disease (AD) models have shed some light on the involvement of eIF2B activation in LTD. Amyloid-β oligomer (AβO) accumulation is an age-related pathological hallmark of AD triggering ATF4-dependent neuronal cell death, resulting in progressive cognitive decline [137]. ISRIB ameliorated AβO-induced cognitive deficiency in rodents, is potentially linked to loss of eIF2B content observed in *post-mortem* AD brains [138]. Surprisingly, two other studies failed to recapitulate the beneficial cognitive effects of eIF2B activation in AD mice models [139,140]. Although the authors suggest different administration regimens and absence of ISR markers as causative reasoning for these unexpected results, AβO accumulation has been previously reported to selectively elevate LTD [141], favouring the p-eIF2α-dependent axis of synaptic plasticity. In support of this, eIF2B activation prevented proper object-placing learning of healthy rodents, which requires p-eIF2α-dependent translation [129]. Accordingly, augmenting p-eIF2α corrected deficient LTD in dystonia mice models [142]. Therefore, tailoring eIF2B function depending on the level of dependence of p-eIF2α could offer new avenues of therapeutic interventions.

Potential roles of eIF2B PTM modulation in cognition also warrants further investigation. A recent report has shown a novel role for eIF2B modulation during axonal wiring [143]. Rapid protein synthesis in growing axons overloads the ER, alleviated by eIF2α phosphorylation which paradoxically prevents key bursts of global translation. Guidance-cue Sema3A signalling overcomes this constraint by transiently supressing GSK-3β-mediated phosphorylation of eIF2Bε (Ser535), enhancing eIF2B activity to rescue global translation over specific time courses [143]. Additionally, lithium treatment in Down syndrome rodent models has been shown to inhibit GSK-3β activity [144], and thereby increase eIF2B activity, and improve synaptic strength [145].

Recent studies suggest that normal cognition relies on eIF2α-dependent translation within specific neuronal subtypes. Learning tasks in mice models reduced p-eIF2α in specific subsets of excitatory and inhibitory neurons [146]. Similarly, selective manipulation of PERK-eIF2α signalling in dopaminergic neurons resulted in multiple cognitive failures [147]. Additionally, conflicting reports highlight the need to address the involvement of other cell types in cognitive decline. Growth factor BDNF has been shown to up-regulate ATF4 mRNA translation independently of p-eIF2α in hippocampal neurons [148], whilst others report enhanced eIF2B activity upon BDNF treatment in similar cultured models [149]. It would be worthwhile to investigate how eIF2B activation within different cell types such as microglia, astrocytes and oligodendrocytes are involved in cognitive decline.

## Mutations in eIF2B linked to vanishing white matter disease (VWMD)

The importance of eIF2B function within the cell is highlighted by the fact that loss of function mutations in any of the five subunits of eIF2B lead to the fatal neurological disorder, leukoencephalopathy with vanishing white matter (VWMD), also known as childhood ataxia with central nervous system hypomyelination (CACH) [7]. Although eIF2B is a global regulator of translation, glial cells appear selectively vulnerable, with astrocytes playing a central role in VWMD pathophysiology [150,151]. Patients suffer chronic degradation of the cerebral white matter associated with increased numbers of oligodendrocyte precursor cells and immature astrocytes [7]. In classical cases, disease onset occurs in childhood and is symptomatically characterised by cerebellar ataxia, spasticity, mild mental decline. Less commonly, patients may also present with ovarioleukodystrophy, loss of vision and epilepsy, and in very rare cases multiorgan defects have been observed [7]. However, phenotypically, symptoms and disease progression vary dramatically, likely due to the genetic complexity of VWMD. Inheritance is autosomal recessive, and mutations are most commonly missense, existing in homozygous or heterozygous states [152], and close to 200 mutations have been described in the Human Genome Database (www.hgmd.cf.ac.uk). Currently, no treatment is available for this disease although promising therapeutics have emerged, including ISRIB, 2BAct and Guanabenz, the latter of which is currently being used as part of the first VWMD clinical trials [76,153,6,154].

**Figure 6:**
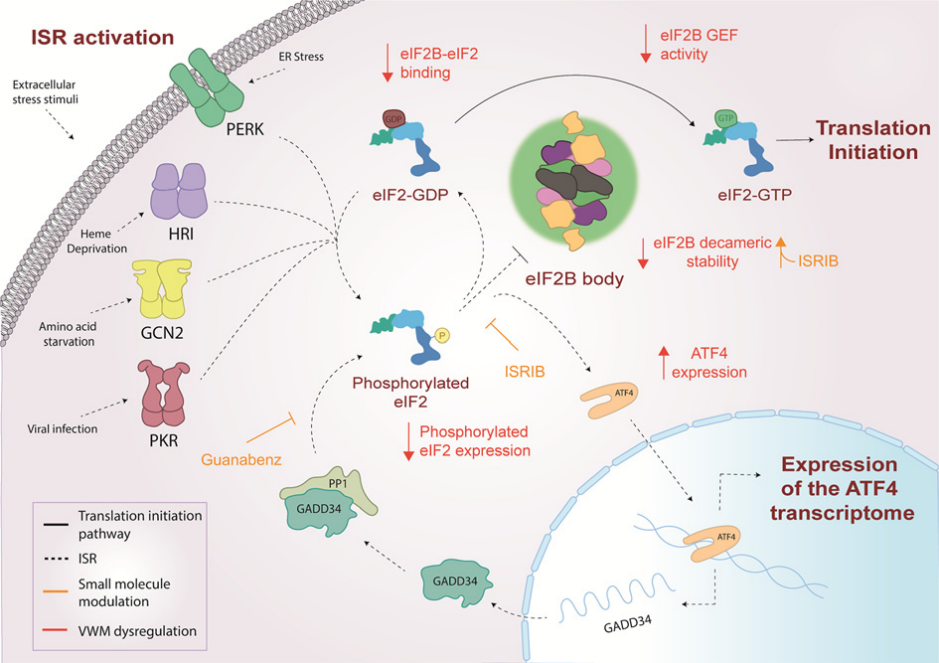
Integrated stress response (ISR) dysregulation and modulation by small molecules in VWMD In response to various cellular stress stimuli, eIF2α kinases (PERK, HRI, GCN2, PKR) are selectively activated and phosphorylate eIF2α, resulting in the inhibition of eIF2B GEF activity and suppression of global translation. The translation of ISR effector mRNAs such as ATF4 is up-regulated promoting stress response gene expression. If stress is overcome GADD34 promotes dephosphorylation of eIF2α restoring homeostatic translation. VWM mutations can decrease eIF2B GEF activity, complex formation or eIF2B body formation, resulting in ISR dysregulation including increased ATF4 expression and decreased eIF2α phosphorylation. ISRIB and Guanabenz modulate ISR signalling through increasing eIF2B complex formation and decreasing dephosphorylation of eIF2α respectively, highlighting therapeutic potential for the treatment of VWM.

While the genetic link between eIF2B and VWMD is clear, the precise contributing role of mutated eIF2B in the progression of VWMD remains elusive. Biochemical analyses have investigated the functional effects that VWMD mutations have on eIF2B. Some mutations destabilise interactions between eIF2B subunits affecting complex formation, whereas other mutations can affect the GEF activity of eIF2B or the modulation of this activity by p-eIF2α [155–160] (Figure 6). The recent discovery of ISRIB is a promising avenue in the treatment of VWMD caused by mutations that destabilise the decameric conformation of eIF2B [75,34,35] (Figure 6). *In vitro* analyses of VWMD mutations associated with decreased eIF2B decameric stability, have demonstrated that ISRIB can rescue the stability of the eIF2B decamer and subsequently the GEF activity (Figure 6) [75].

However, VWMD mutations have been identified that have no impact on the biochemically characterised *in vitro* functions of eIF2B but cause some of the most severe forms of disease [160,161], suggesting that eIF2B has functions *in vivo* that are not accounted for *in vitro*. Recently, two studies have investigated the therapeutic potential of ISRIB, and its derivative 2BAct, in VWMD mouse models, homozygous for eIF2Bε^R191H^ (R195H in humans) (2B5^ho^ mice) or homozygous for eIF2Bδ^R484W^ (R483W in humans) and heterozygous for eIF2Bε^R191H^ (2B4^ho^2B5^he^ mice) [76,6]. *In vitro* studies have shown both eIF2Bε^R195H^ and eIF2B^R483W^ mutations are associated with decreased decameric stability and eIF2B GEF activity, which can be rescued by ISRIB [161,75]. Clinical signs of VWMD were normalised in 2BAct treated 2B5^ho^ mice and ISRIB treated 2B4^ho^2B5^he^ mice; although clinical symptoms and pathology were significantly improved in ISRIB treated 2B5^ho^ mice, mild signs of neurological deterioration were still observed [76,6]. Therefore, these data suggest that other *in vivo* factors could influence ISRIB’s effect. In order for ISRIB to act as an eIF2B activator, a pool of unassembled eIF2B tetramers must be available [1]. Hodgson et al*.* identified the presence of eIF2B bodies with varying degrees of eIF2B subunits in mammalian cells [107]. Additionally, it has been shown that VWMD mutations can impact upon the integrity and functionality of eIF2B bodies in yeast model systems [99,104], highlighting a possible involvement of localised pools of eIF2B in VWMD pathology. Deletion of eIF2Bα causes dispersal of eIF2B bodies. It could therefore be hypothesised that ISRIB could rescue eIF2B body formation in the presence of these VWMD mutations that disrupt body formation. However, mutational defects which impact upon catalytic function disrupt eIF2B body integrity, and it is currently unclear whether ISRIB’s ability to enhance eIF2B catalytic function would be sufficient to rescue localisation in the case of these mutants. Coupling *in vitro* analysis with in-cell assays could provide further insight into the influence of ISRIB on specific VWMD mutations.

VWMD mutations have also been found to dysregulate the ISR (Figure 6), and whilst the impact of VWMD mutations on eIF2B structure and function are diverse, dysregulation of the ISR appears to be a common consequence. Increased expression of the ATF4 driven transcriptome has been observed in a VWMD mouse model, homozygous for eIF2Bε^R191H^ (R195H in humans), and in VWMD patient brain samples [76,6]. Characteristic of a chronic ISR response, increased levels of p-eIF2α were not observed, likely explained by an increased GADD34 expression. Authors suggest that decreased eIF2B GEF activity caused by VWMD mutations is the driving force behind ISR induction. The ATF4 transcriptome is unable to restore homeostatic levels of eIF2B GEF activity thus inducing chronic stress. These findings have highlighted Guanabenz as a promising therapeutic for VWMD. Guanabenz is a potent agonist of the α2-adrenergic receptor and indirectly regulates eIF2B activity, by targeting the activity of the eIF2α phosphatase, GADD34, prolonging eIF2α phosphorylation during cellular stress, thereby decreasing the burden of global translation allowing more energy for homeostatic reprogramming [162] (Figure 6). However, guanabenz has also been shown to impact protein trafficking in the absence of any changes to eIF2α phosphorylation [163]. While the exact mode of action of guanabenz with respect to VWMD is unclear, administration of Guanabenz to VWMD mice (homozygous for eIF2Bε^R191H^) has been shown to improve clinical symptoms of VWMD [153] and is now undergoing clinical trials [154].

Interestingly, in both VWMD mouse and patient brain, dysregulation of the ISR was observed in astrocytes and myelinating oligodendrocytes [76,6]. *In vitro studies* have highlighted that in the presence of VWMD mutations, specific cell-types appear more vulnerable to episodes of acute stress. These cells exhibit a heightened stress response, characterised by a hyper-induction of ATF4 and hyper-suppression of translation [75,164–167]. Chronic expression of ATF4 has also been observed in mouse models of mitochondrial dysfunction, and mutations in mitochondrial proteins have been associated with leukoencephalopathies [168–175]. Interestingly, transcriptome and proteome datasets generated from embryonic fibroblasts and whole brains of VWMD eIF2Bε^R132H^ mice (R136H in humans) have highlighted an impaired mitochondrial phenotype [176]. Although eIF2B does not regulate translation of the mitochondrial genome directly, the mitochondrial protein synthesis machinery itself is cytoplasmically translated and thus under the control of eIF2B. Fibroblast, oligodendrocyte precursor and astrocyte cells from the eIF2Bε^R132H^ mouse model suffer impairment of oxidative phosphorylation and ATP production [177]. While an adaptive increase in mitochondrial abundance restores this decreased oxidative phosphorylation in fibroblasts, increased mitochondrial abundance cannot rescue this phenotype in astrocytes or oligodendrocyte precursor cells [177,178]. VWMD highlights the dependence of glial cells on the precise accuracy of eIF2B mediated translational control.

## eIF2B mutations and their link to neonatal diabetes

**Figure 7:**
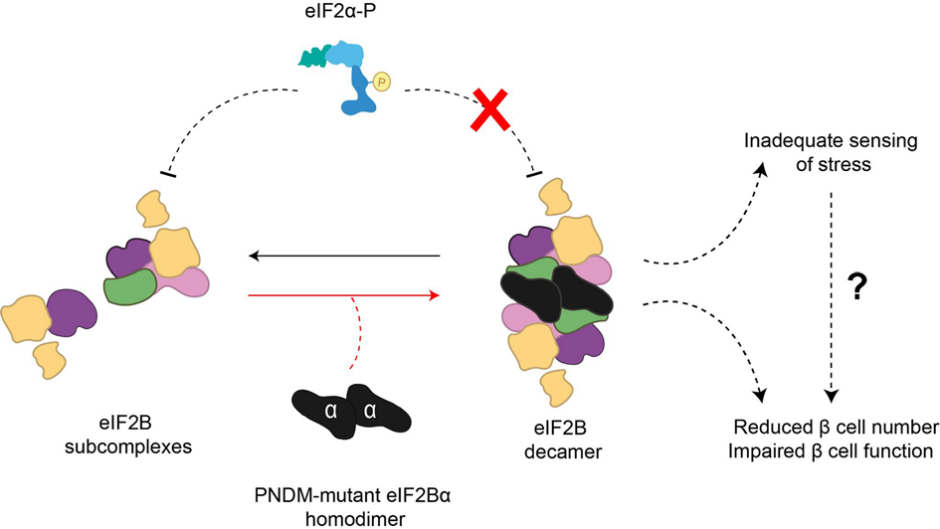
eIF2Bα PNDM mutations may lead to inadequate stress sensing and ISR in pancreatic β cells eIF2Bα missense mutations that lead to PNDM are present in residues which interact with eIF2 or the binding surface of p-eIF2α. Additionally, yeast studies have found that specific PNDM mutations can also disrupt eIF2B bodies and impact eIF2B decameric formation. This suggests that p-eIF2α binding to eIF2B is impeded, indicating a hindrance in stress-sensing which may lead to pancreatic β cell dysfunction or reduced number.

Recently, an increased incidence of heterozygous *de novo* missense variants in the EIF2B1 gene, which encodes eIF2Bα has been identified in patients with permanent neonatal diabetes mellitus (PNDM), a disorder resulting in early onset diabetes, typically diagnosed within the first 6 months of life [179]. *In silico* protein analysis revealed that the eIF2Bα missense variations identified in PNDM patients were present in either the binding surface occupied by p-eIF2α or altered residues involved in the interaction with eIF2 (Figure 7) [175]. Mutations in eIF2Bα have been previously characterised in the yeast orthologue of eIF2Bα, Gcn3p, and were categorised as either general control non-derepressible (*gcn^−^* mutations), which affects the regulatory activity of eIF2B, or as general control de-repressible (*gcd*^−^ mutations), which affects the catalytic activity of eIF2B [27]. Interestingly two of the mutations identified in PNDM involve residue 44 on eIF2Bα. Mutations in this residue have previously been analysed in yeast and classified as *gcn*^−^ with reduced sensitivity to p-eIF2α [17]. This suggests that the PNDM eIF2Bα mutations may hinder or impede the binding between eIF2Bα and p-eIF2α, thus leading to an inadequate sensing and response to cellular stress (Figure 7).

Additionally, in yeast, mutations in eIF2Bα^E44^ also compromise the formation of eIF2B bodies [99], either by the decrease of eIF2Bα levels, leading to a decameric instability or by destabilising eIF2B subunit binding. It is known that for the recognition and binding of p-eIF2α, complete eIF2B decamer complex configuration is essential [36,37,180]. As such, the faulty sensing of stress in PNDM mutations may be also linked to the incorrect formation of eIF2B bodies. Furthermore, the assembly of these bodies may play an important role in the regulatory function of this complex.

When juxtaposed with eIF2Bα mutations associated with VWMD, the sites of PNDM eIF2Bα mutations differ greatly. The VWMD mutations are predominantly located in the C-terminal of eIF2Bα and appear to disrupt the formation of the eIF2B decamer, whereas PNDM mutations occur within the N-terminal region and appear to disrupt activation of the ISR [37,179]. However, interestingly in a VWMD patient that exhibited diabetic ketoacidosis, the homozygous missense variant was present in the N-terminal (eIF2Bα^L49R^) [181], which would suggest a similar eIF2Bα defect observed in PNDM mutations and could explain the diabetic presentation.

While PNDM associated with heterozygous eIF2Bα variants do not exhibit severe neurological features, two reported cases displayed mild learning disability or attention deficit disorder [181], highlighting the link between cognitive abilities and eIF2B, which was discussed previously.

The most common monogenic PNDM subtype is Wolcott-Rallison syndrome (WRS; OMIM 226980) and is caused by recessive missense and truncation mutations in the EIF2AK3 gene, which encodes the eIF2 alpha kinase, PERK [182–185]. It is thought that since these mutations inhibit activation of the ISR the cells are unable to appropriately respond to stress and this ultimately leads to β-cell death, an analogous consequence of the eIF2Bα loss of function mutations. Interestingly, while β-cell death was not observed in knockout animal models of HRI [186], PKR [187] and GCN2 [188], PERK^−/−^ mutants displayed degenerated pancreatic β-cells coupled with deficient function, leading to the development of diabetes mellitus [189].

High levels of glucose have been shown to lead to an increase of eIF2B activity and an increase in insulin protein synthesis in pancreatic β-cells [190]. Additionally, acute to moderate intensity resistance exercise has been shown to increase rates of protein synthesis and eIF2B activity in non-diabetic and moderately diabetic animal models. However, in severe insulin deficiency models this increase in eIF2B activity is inhibited and consequently, decreased levels of protein production are maintained [191,192]. GSK3 inhibition has been linked to insulin-stimulated liver glycogen synthesis [193], leading to eIF2Bε Ser535 dephosphorylation and eIF2B activation. This mechanism would account for the lack of eIF2B activation in severe hypoinsulinemia. Taken together, there is a clear direct association between eIF2B and pancreatic β-cell function and physiology.

Given the essential roles that PERK activation and eIF2B regulation play in β-cells, therapeutic targets that may lead to an abatement of stress, i.e., key factors in these stress pathways or rescue of function of those same factors, are potential and exciting approaches to restore β-cell health in diabetes. Therefore, targeting eIF2B could be a novel and effective avenue for therapy in β-cell disorders, such as PNDM or even diabetes mellitus [194].

ISRIB stabilises the productive conformation of decameric eIF2B, thus concurrently promoting eIF2B GEF activity and attenuating ISR effects [71,34,35]. However, the impact of the PNDM-associated eIF2Bα variants in the formation and localisation of eIF2B bodies, and the resulting impact on the GEF activity in mammalian cells is still unknown. Future studies concerning these areas may define the role of eIF2B in β-cells and in PNDM, and possibly open new therapeutic opportunities, such as ISRIB.

## Conclusions and future directions

eIF2B is recognised as a critical control point in the regulation of translation. The inhibition of eIF2B GEF activity by p-eIF2α serves as the core event of the ISR, coordinating cellular responses to a vast range of cellular stresses. Dysregulation of eIF2B GEF activity and the ISR is now well established in a number of diseases and small molecules that target eIF2B have been shown to alleviate this dysfunction in many disease situations. Recent structural developments have provided extensive knowledge of the mechanisms that control eIF2B GEF activity and its inhibition by p-eIF2α, enhancing our understanding of how eIF2B mutations impact the ISR and the mechanism through which small molecules, such as ISRIB, can modulate eIF2B activity.

An important consideration for future research is the impact of cell-type specificity on eIF2B GEF activity and ISR regulation. It is clear that different cell types are selectively vulnerable to certain conditions of cellular stress and that mutations in specific ISR genes can cause detrimental effects in certain cell types. Uncovering the mechanism by which specific cells can activate the ISR will advance our molecular understanding of the ISR in disease. In-cell analysis of eIF2B has demonstrated the presence of eIF2B bodies; accumulations of functional eIF2B at specific foci within the cell cytoplasm. The eIF2B subunit composition of eIF2B bodies is suggested to influence GEF activity and is regulated by the ISR and the small molecule ISRIB. Characterisation of eIF2B bodies in different cell types could highlight cell-type specific dependencies for threshold levels of eIF2B GEF activity and regulation during the ISR as well as identifying new eIF2B protein–protein interactors that may influence function and body formation. The association of specific binding factors to eIF2B and the role of post translational modifications on eIF2B subunits may also provide critical information regarding cell specific phenotypes and response mechanisms.

## Abbreviations

ATF4: activating transcription factor 4
CHOP: C/EBP homologous protein
eIF2: eukaryotic initiation factor 2
eIF2B: eukaryotic initiation factor 2B
ER: endoplasmic reticulum
GADD34: growth arrest and DNA damage inducible protein
GCN2: general amino acid control nonderepressible 2
GEF: guanine nucleotide exchange factor
HRI: heme-regulated inhibitor
ISR: integrated stress response
ISRIB: integrated stress response inhibitor
LTD: long-term depression
LTP: long-term potentiation
NTD: N-terminal domain
PERK: protein kinase RNA-like endoplasmic reticulum kinase
PKR: protein kinase R
PNDM: permanent neonatal diabetes mellitus
PTM: post-translational modification
uORF: upstream open reading frame
VWMD: vanishing white matter disease
